# Supplementary material for: Production of gas-releasing electrolyte-replenishing Ah-scale zinc metal pouch cells with aqueous gel electrolyte
Source: Nat Commun. 2023 Jul 14;14:4211. doi: 10.1038/s41467-023-39877-5 (PMC10349122; doi:10.1038/s41467-023-39877-5)
Supplement: Supplementary file 1 — Suplemantary information [file 41467_2023_39877_MOESM1_ESM.pdf]

Supplementary Information

Production of gas-releasing electrolyte-replenishing Ah-scale zinc metal pouch cells with aqueous gel electrolyte

Feifei Wang[1,2,3,4], *Jipeng Zhang*[1,3], *Haotian Lu*[1,2,3,4], *Hanbing Zhu*[1,3], *Zihui Chen*[1,3], *Lu Wang*[1,2,3,4], *Jinyang Yu*[1,3], *Conghui You*[2], *Wenhao Li*[5], *Jianwei Song*[5], *Zhe Weng*[1,3], *Chunpeng Yang*[1,3*], *and Quan-Hong Yang*[1,2,3*]

[1]Nanoyang Group, Tianjin Key Laboratory of Advanced Carbon and Electrochemical Energy Storage, School of Chemical Engineering and Technology, National Industry-Education Integration Platform of Energy Storage, and Collaborative Innovation Center of Chemical Science and Engineering, Tianjin University, Tianjin 300072, China

[2]Joint School of National University of Singapore and Tianjin University, International Campus of Tianjin University, Fuzhou 350207, China

[3]Haihe Laboratory of Sustainable Chemical Transformations, Tianjin 300192, China

[4]Department of Chemistry, National University of Singapore, Singapore 117543, Singapore

[5]State Key Laboratory for Strength and Vibration of Mechanical Structures, Xi'an Jiaotong University, Xi'an 710049, China

E-mail: cpyang@tju.edu.cn, qhyangcn@tju.edu.cn

Supplementary Figures

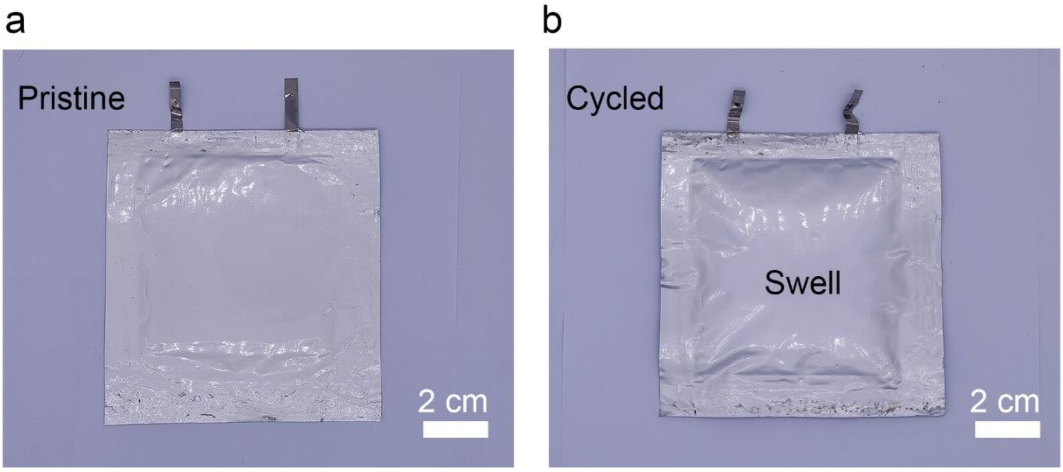

Supplementary Fig. 1 | Optical image of a symmetric pouch cell (**a**) before and (**b**) after cycling. The distinct battery swell is observed after cycling because of the HER (Zn symmetric cells: 10 mA cm^{-2} with charge/discharge capacity of 10 mAh cm^{-2} for 50 cycles at 25 °C, pressure: 370 kPa,).

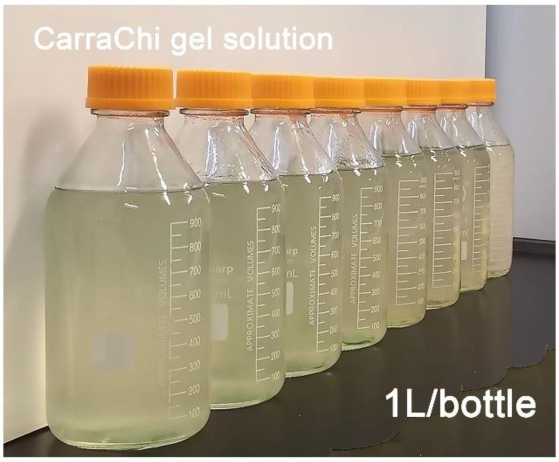

Supplementary Fig. 2 | Large-volume CarraChi gel solutions produced by stirring.

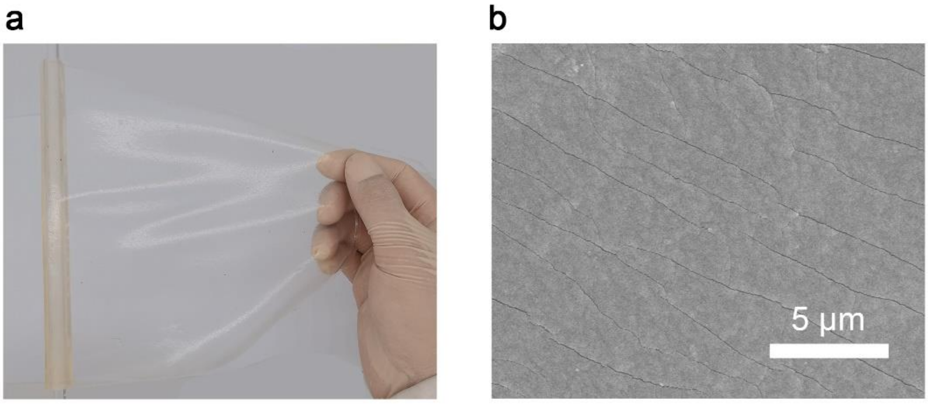

Supplementary Fig. 3 | a, Optical and **b,** SEM images of the CarraChi membrane.

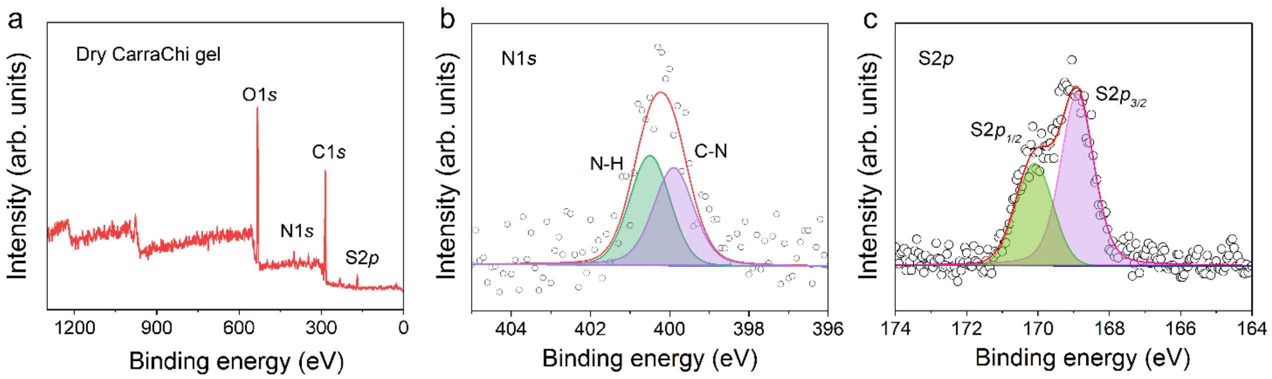

Supplementary Fig. 4 | XPS measurements and analyses of the dry CarraChi gel: a, full spectra and **b**, N1*s* spectra, and **c**, S2*p* spectra.

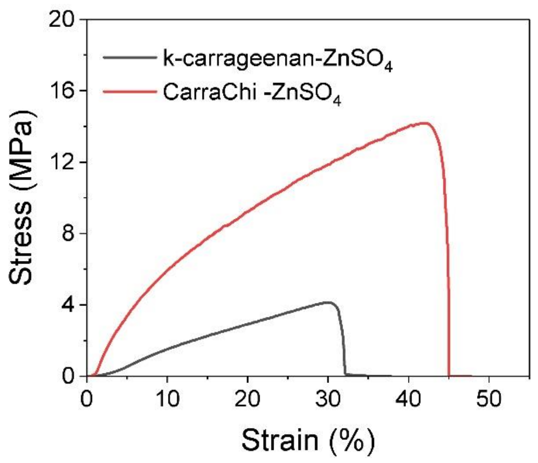

Supplementary Fig. 5 | Stress-strain curves of the CarraChi-$ZnSO_4$ and k-carrageenan-$ZnSO_4$.

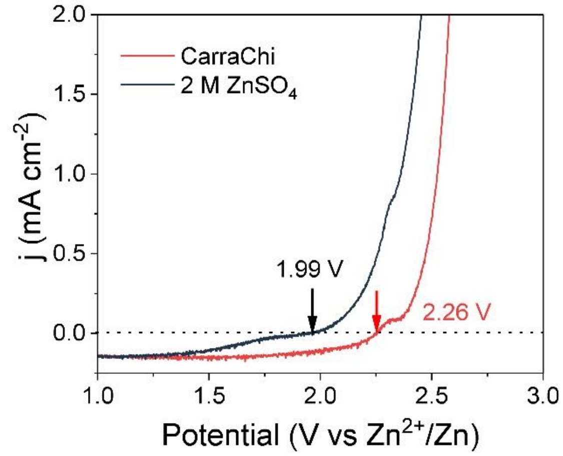

Supplementary Fig. 6 | The electrochemical window of the CarraChi gel electrolyte and 2 M $ZnSO_4$ (25 °C).

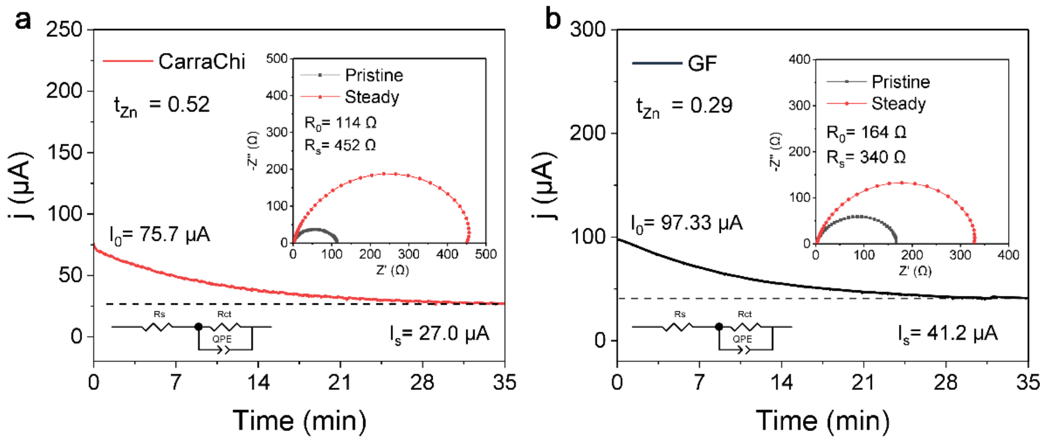

Supplementary Fig. 7 | Chronoamperometry curves with a constant voltage polarization of 20 mV (measured at 25 °C). The insets show the EIS curves and the equivalent circuits before and after polarization. **a**, Zn|CarraChi|Zn; **b**, Zn|GF|Zn (Fitted results are listed in Supplementary Table 3).

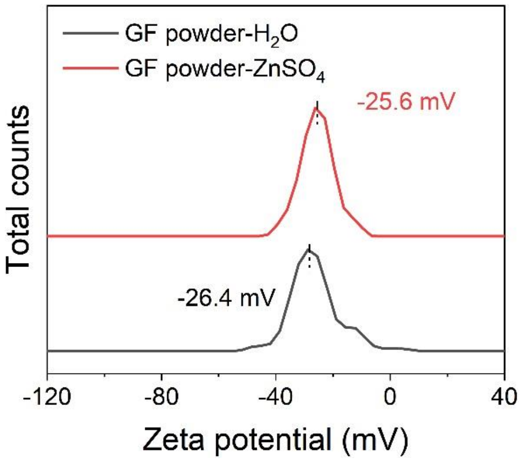

Supplementary Fig. 8 | Zeta potential: the pulverized GF powder in an aqueous solution before and after the addition of $ZnSO_4$.

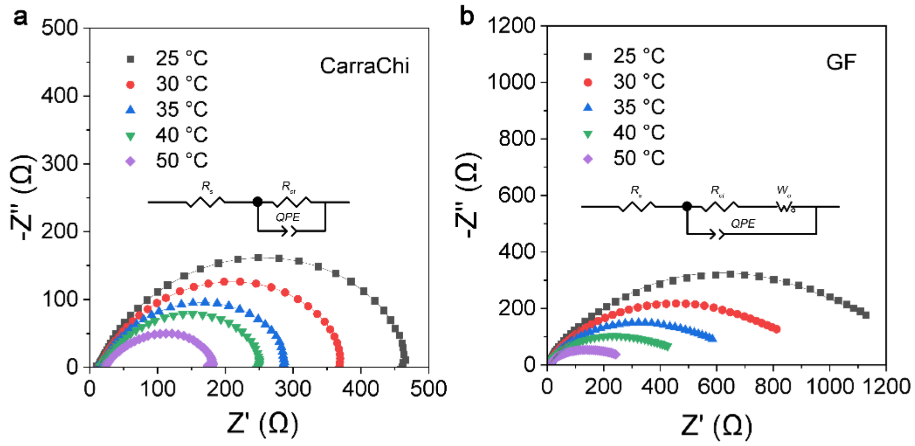

Supplementary Fig. 9 | EIS curves and equivalent circuits of the Zn symmetric cells at temperatures from 25 to 50 °C. **a**, with the CarraChi gel electrolyte; **b**, with the GF separator. (The fitted R_{ct} values are listed in Supplementary Tables 4 and 5).

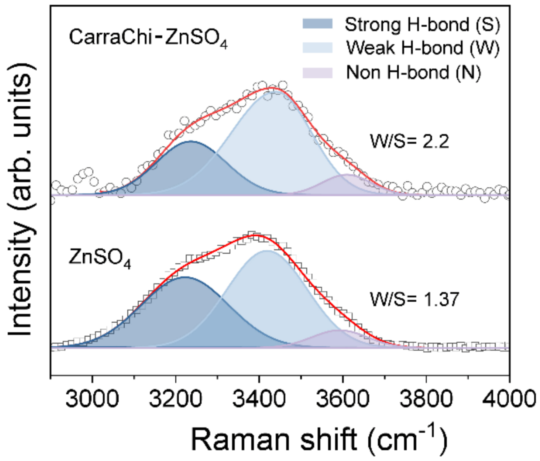

Supplementary Fig. 10 | Raman spectra of $ZnSO_4$ solution, and CarraChi gel electrolyte (measured at 25 °C).

The wide peak from 3000 to 3700 cm^{-1} is due to the O-H stretching vibration of water molecules, which can be divided into three components according to the literature.[1] The major peaks at ~3230, ~3450, and ~3620 cm^{-1} correspond to water molecules with strong (S), weak (W), and Zn^{2+}-solvated water (non H-bonds, N), respectively.

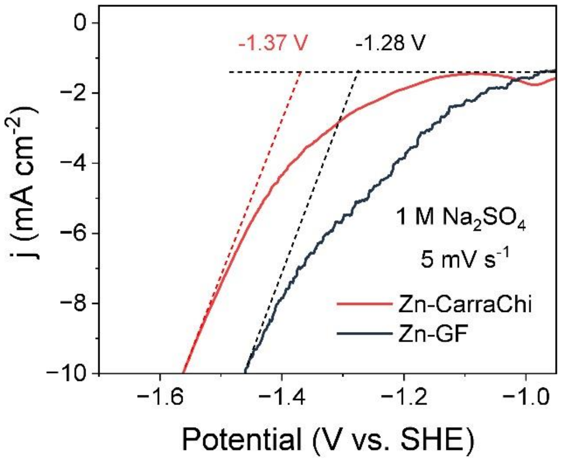

Supplementary Fig. 11 | LSV curves of Zn with CarraChi and GF in coin cells (25 °C).

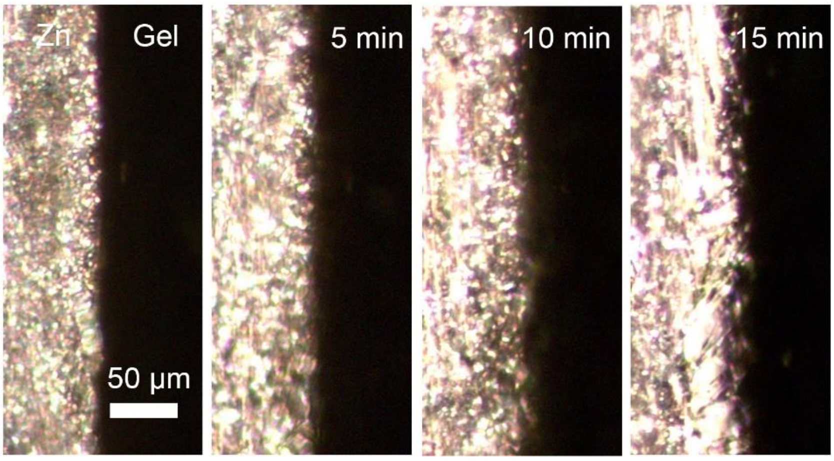

Supplementary Fig. 12 | In situ optical microscope images of Zn foil with CarraChi during plating at 10 mA cm^{-2} in an open electrolytic cell (25 °C), without finding H_2 bubbles.

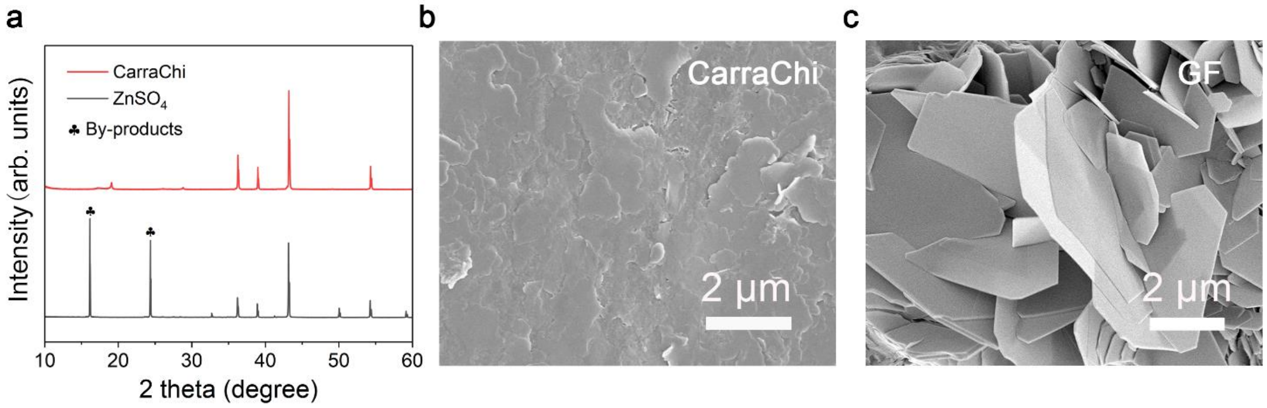

Supplementary Fig. 13 | a, XRD patterns of Zn foil soaked with CarraChi and $ZnSO_4$ solution. SEM images of the Zn foil after 1 week of direct contact: **b**, with the CarraChi, and **c,** with the GF separator.

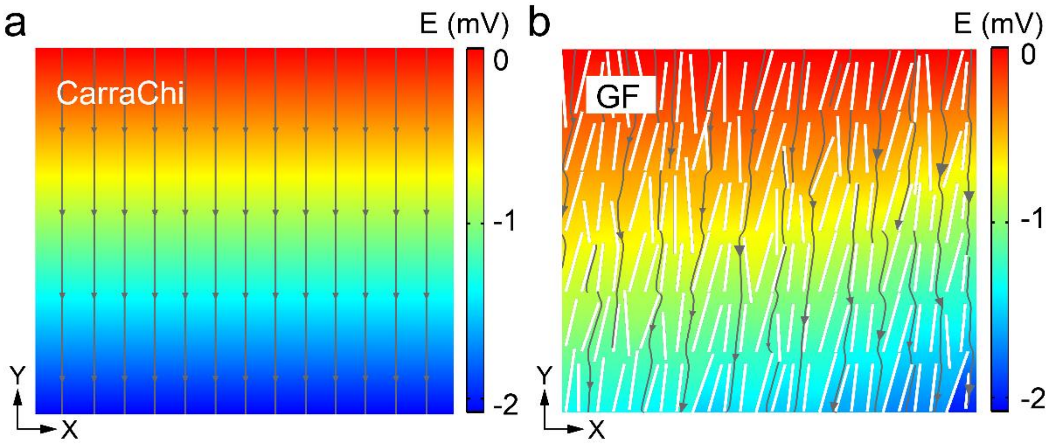

Supplementary Fig. 14 | Simulated electric potential and electric current distribution for **a**, Zn|CarraChi|Zn and **b**, Zn|GF|Zn symmetric cells at 10 mA cm^{-2} (The thin strips represent glass fiber, and E represents potential, mV).

The Zn anode with CarraChi exhibits homogenized Zn^{2+} concentration along X-axis, which facilitates uniform Zn^{2+} supplement during the Zn deposition process. In contrast, a higher concentration gradient is observed along X-axis for the Zn anode with GF separator, making it easier for Zn dendrites growth at the Zn/GFA interface (Fig. 3d, e). When the potential is applied, a much more uniform electric field distribution is achieved along the X-axis with the CarraChi gel electrolyte, manifesting the effective suppression of Zn dendrite during cycling.

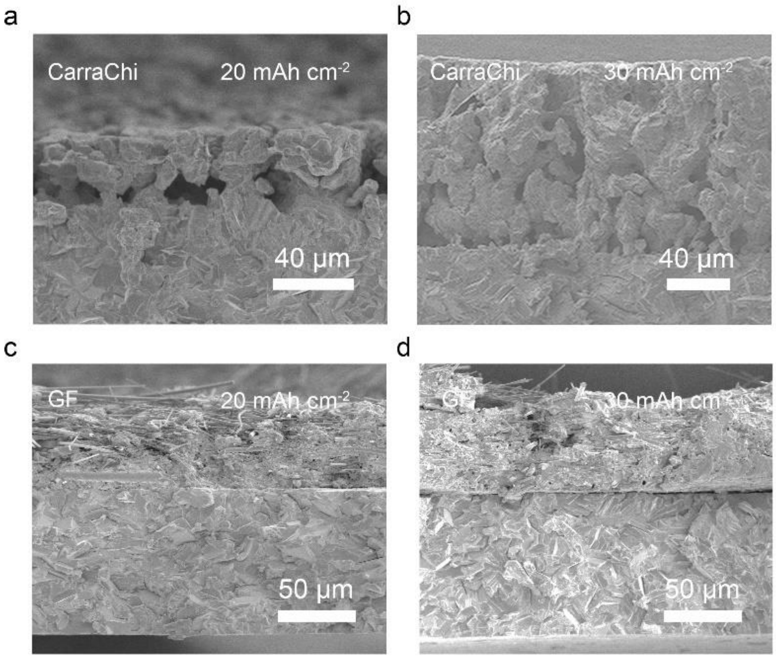

Supplementary Fig. 15 | a-d, SEM images of bare Zn deposited at 10 mA cm^{-2} with different capacities in coin cells (25 °C). **a**, 20 mAh cm^{-2} and **b**, 30 mAh cm^{-2} in Zn|CarraChi|Zn; **c**, 20 mAh cm^{-2} and **d**, 30 mAh cm^{-2} in Zn|GF|Zn.

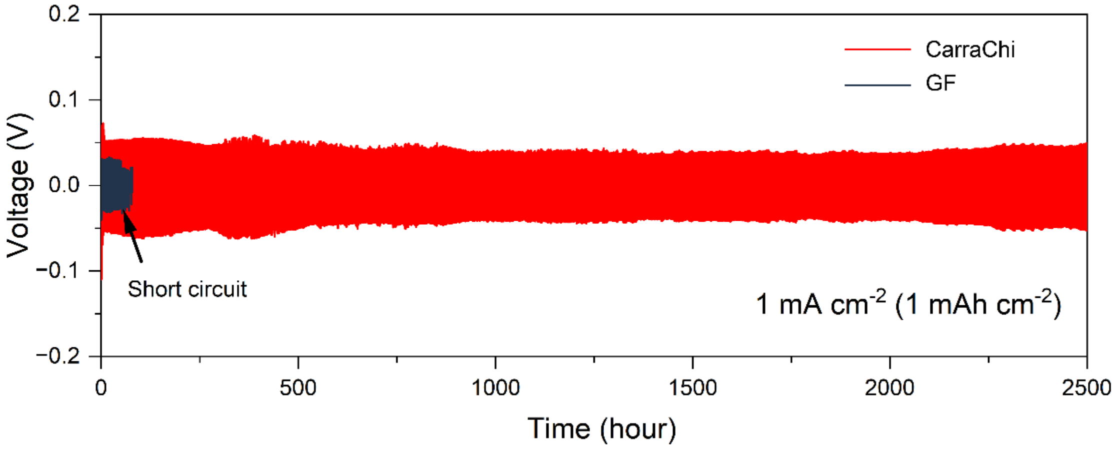

Supplementary Fig. 16 | Cycling stability of Zn symmetric cells with CarraChi and GF (1 mA cm^{-2} with 1 mAh cm^{-2}) in coin cells (25 °C).

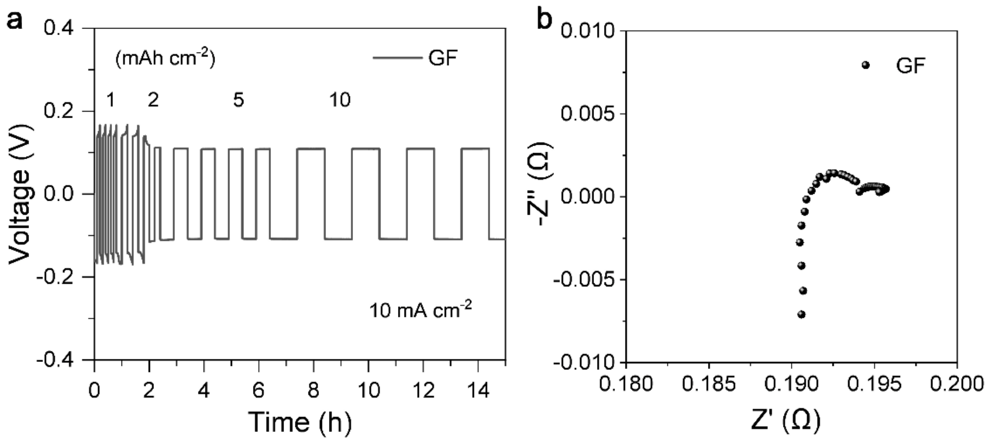

Supplementary Fig. 17 | a, Cycling stability of the symmetric Zn|GF|Zn pouch cell with various capacities from 1 to 10 mAh cm^{-2} at 10 mA cm^{-2}. **b**, EIS curve of the Zn|GF|Zn pouch cell after cycling.

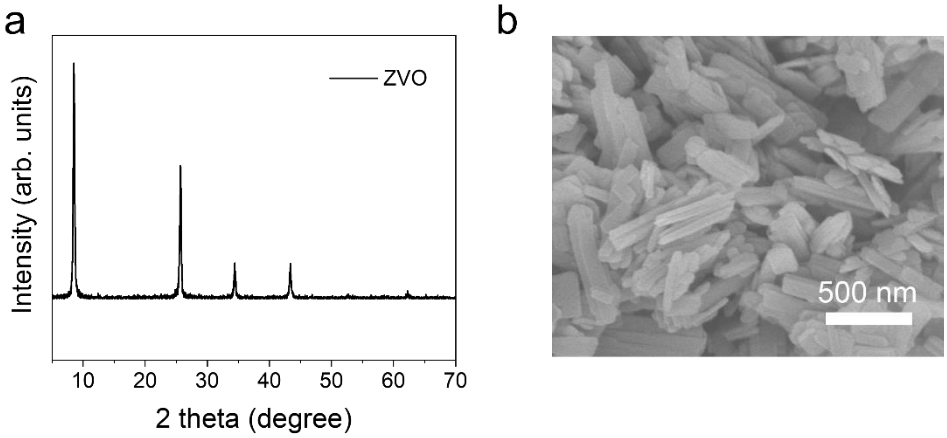

Supplementary Fig. 18 | a, XRD pattern and **b,** SEM image of the prepared ZVO nanorods before electrode formulation.

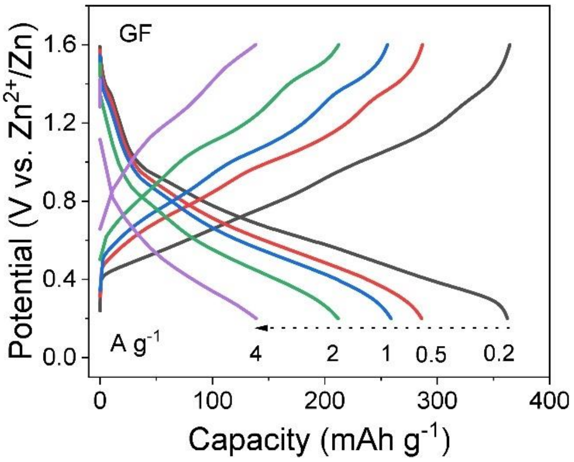

Supplementary Fig. 19 | Voltage profiles of Zn|GF|ZVO at various charge/discharge specific currents in coin cells at 25 °C (based on active ZVO mass).

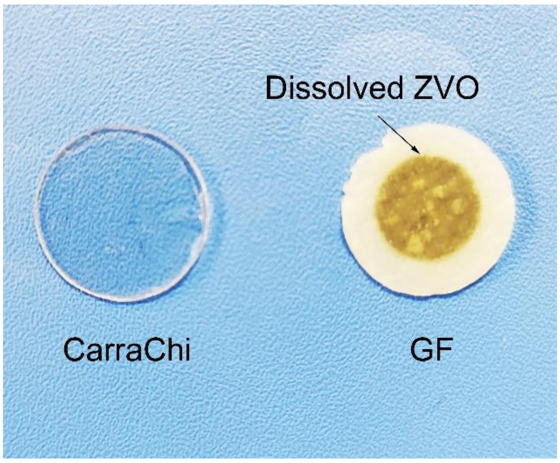

Supplementary Fig. 20 | Optical images of CarraChi and GF after 1 hour of direct contact with ZVO cathode in the air (The dissolved ZVO absorbed on GF while there is no dissolved ZVO on CarraChi).

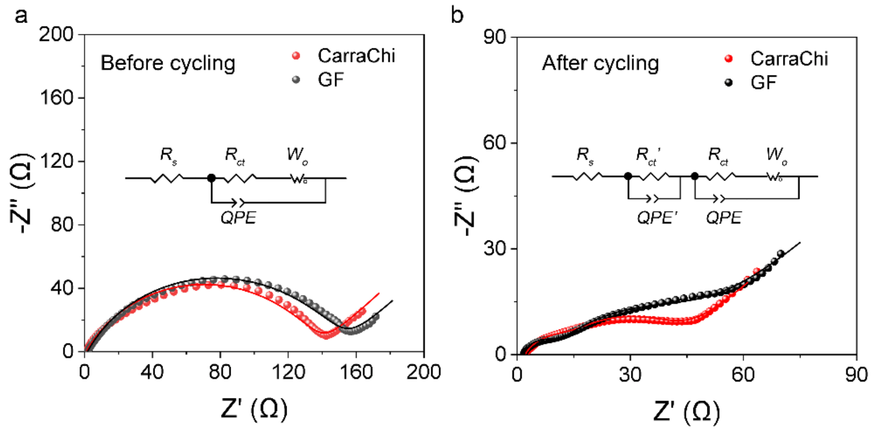

Supplementary Fig. 21 | EIS curves and equivalent circuits of Zn|CarraChi|ZVO and Zn|GF|ZVO in coin cells (25 °C). a, before cycling, and **b**, after cycling (Fitted R_{ct} values in the equivalent circuit are listed in Supplementary Table 7).

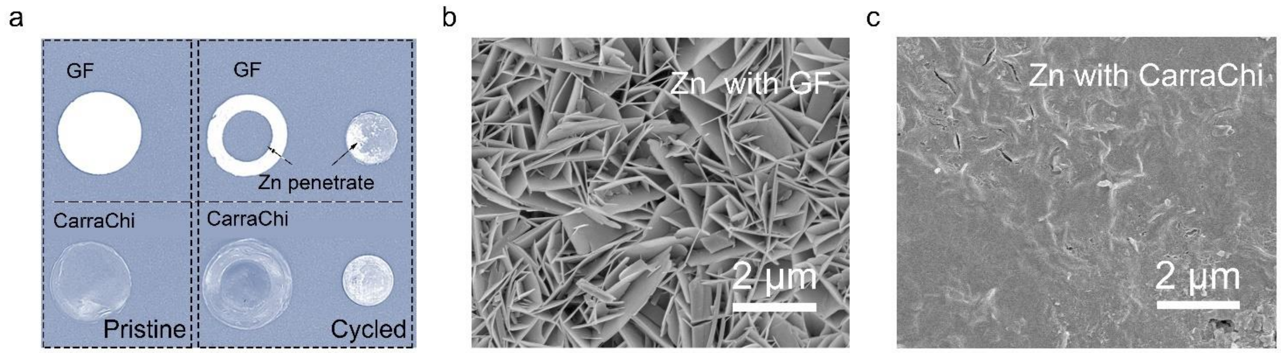

Supplementary Fig. 22 | a, Optical images of the pristine and cycled CarraChi gel, GF, and Zn anode in coin cells (25 °C). SEM images of cycled Zn with **b**, GF separator, and **c**, CarraChi gel electrolyte.

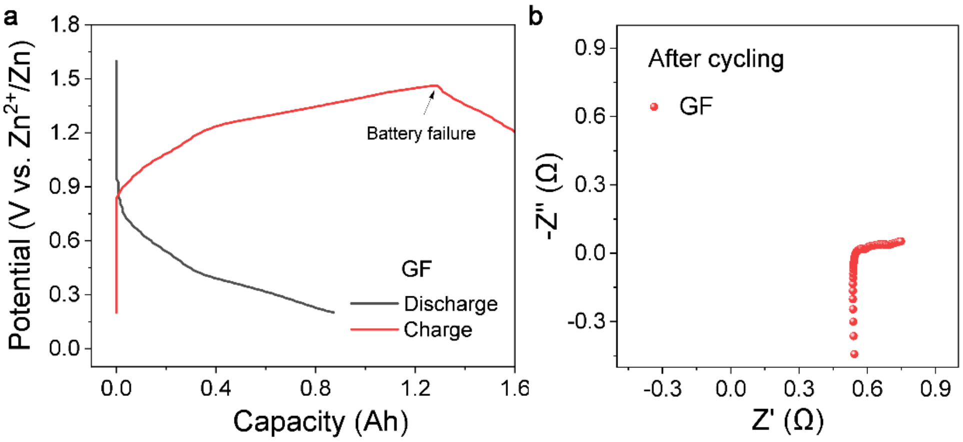

Supplementary Fig. 23 | a, Voltage profiles and **b**, EIS curves of Zn|GF|ZVO pouch cell when the battery fails (25 °C).

Supplementary Tables

Supplementary Table 1 | Comparisons of previously reported gel electrolytes with our work based on the tensile strength, ionic conductivity, Zn^{2+} transference number (t_{Zn}), applied current density (j), capacity, and cycle number.

Gel type	Tensile strength (MPa)	Ionic conductivity (mS cm^{-1})	t_{Zn}	j (mA cm^{-2})	Capacity (mAh cm^{-2})	Cycle number	References
CarraChi	112	5.3	0.52	10	35	574	Our work
CMC/PNiPAM	37.9	0.17	0.54	5	1	-	[2]
P(ICZn-AAm)	0.12	2.15	0.93	0.25	-	-	[3]
PAM-PGO	-	31	-	3.5	1	1000	[4]
PZHE	-	32	0.656	3	-	-	[5]
ILZE	9.12	16.9	-	2	0.5	6000	[6]
SPE	-	19.6	0.7	0.1	0.5	180	[7]
Alg-Zn	-	18.3	0.75	1.77	0.885	270	[8]
PAMPSZn	-	20	0.4	1	1	2250	[9]
TA-SA	-	24.2	0.74	1.13	-	-	[10]
TPU	3.5	19.8	-	10	10	250	[11]
Zn–SHn	-	34	-	10	5	1000	[12]
PASHE	0.028	32.9	0.84	20	10	100	[13]

All these measurements were conducted at 25 °C.

Supplementary Table 2 | Element contents of the CarraChi gel electrolyte from the XPS results.

**Element**	C	O	S	N	Sum
**At%**	63.26	28.62	2.93	5.19	100.00

Supplementary Table 3 | Fitting results of R_{ct} for the Zn symmetric cells before and after the chronoamperometry test in Supplementary Fig. 7.

Samples		R_{ct}	Error (%)
Zn|CarraChi|Zn	Before	114	6.8
	After	452	1.6
Zn|GF|Zn	Before	164	2.0
	After	340.3	1.2

Supplementary Table 4 | Fitting results of R_{ct} at different temperatures for the Zn symmetric cell with CarraChi in Supplementary Fig. 9a.

Temperature	25 ℃	30 ℃	35 ℃	40 ℃	50 ℃
R_{ct} (Ω)	472	381	300	252	165
Error (%)	1.5	1.6	2.0	1.5	2.4

Supplementary Table 5 | Fitting results of R_{ct} at different temperatures for the Zn symmetric cell with GF in Supplementary Fig. 9b.

Temperature	25 ℃	30 ℃	35 ℃	40 ℃	50 ℃
R_{ct} (Ω)	1222	856	590	431	248
Error (%)	4.8	4.9	2.4	4.9	1.9

Supplementary Table 6 | Comparison of the cycling performance of the Zn symmetric cells with previous reports.

Materials	Current density (mA cm^{-2})	Areal capacity (mAh cm^{-2})	Cycle number	Cumulative capacity (Ah)	Average CE (%)	Citation
CarraChi	10	35 (65% depth of discharge)	574	1286	99.5	This work
CWK	5	5	276	1.083	98	[14]
Polyanionic Gel	10	5	1000	3.925	99	[12]
PZHE	3	1	-	0.589	99	[5]
PAMPS-Zn	1	1	2250	1.766	99.3	[9]
PDA coating	30	30	37.5	0.883	99.5	[15]
Glycerol/Acetonitrile gel	0.5	5	150	0.589	99.5	[16]
PZIB gel	7.5	7.5	205	1.207	99.6	[17]
MXene-Graphene	10	1	5250	4.121	99.67	[18]
TEOS-Cellulose	2	1	400	0.628	99.9	[19]
CSAM-C	3	3	250	0.588	100	[20]

All these measurements were conducted at 25 °C.

Supplementary Table 7 | Fitting results of R_{ct} for the full cells before and after the cycling test in Supplementary Fig. 21.

Samples		R_{ct}	Error (%)
Zn|CarraChi|ZVO	Before	136.9	1.7
	After	30.16	1.6
Zn|GF|ZVO	Before	149.8	1.9
	After	45.5	2.97

Supplementary Table 8 | Comparison of the cyclic performance of pouch full cells with previous reports.

Cathode	Electrolyte	Initial discharge capacity (Ah)	Applied current	Cycles	Discharge capacity retention (%)	References
ZVO	CarraChi	0.9	0.2 A g^{-1}	200	84	Our work
VOH	Sulfolane−H_2O	0.2	0.5 A g^{-1}	55	81.6	[21]
ZnVOH	$ZnSO_4$	0.02	0.1 mA cm^{-2}	200	70.7	[22]
$NH_4V_4O_{10}$	$Zn(CF_3SO_3)_2$	0.015	0.4 A g^{-1}	240	83	[23]
$Zn_{0.55}V_2O_4PO_4·3.2H_2O$	$Zn(CF_3SO_3)_2$	0.0065	0.1 A g^{-1}	60	98.5	[24]
HVO	ZOT-H_2O-60% PEG	0.0035	0.5 A g^{-1}	300	81.7	[25]
V_2O_5	Gel	0.017	1.2 mA cm^{-2}	500	87.8	[26]
VOH	DME-$Zn(OTF)_2$	0.062	-	100	83.5	[27]

All the anodes used in the table are bare Zn foil. All these measurements were conducted at 25 °C.

Supplementary references

1. Zhou X, Zhao F, Guo Y, Rosenberger B, Yu G. Architecting highly hydratable polymer networks to tune the water state for solar water purification. *Sci. Adv.* **5**, eaaw5484 (2019).
2. Dueramae I, Okhawilai M, Kasemsiri P, Uyama H, Kita R. Properties enhancement of carboxymethyl cellulose with thermo-responsive polymer as solid polymer electrolyte for zinc ion battery. *Sci. Rep.* **10**, 12587 (2020).
3. Chan CY, Wang Z, Li Y, Yu H, Fei B, Xin JH. Single-Ion Conducting Double-Network Hydrogel Electrolytes for Long Cycling Zinc-Ion Batteries. *ACS Appl. Mater. Interfaces* **13**, 30594-30602 (2021).
4. Abbasi A, *et al.* Phosphonated graphene oxide-modified polyacrylamide hydrogel electrolytes for solid-state zinc-ion batteries. *Electrochim. Acta* **435**, 141365 (2022).
5. Leng K, *et al.* A Safe Polyzwitterionic Hydrogel Electrolyte for Long-Life Quasi-Solid State Zinc Metal Batteries. *Adv. Funct. Mater.* **30**, 2001317 (2020).
6. Ma LT, *et al.* Hydrogen-Free and Dendrite-Free All-Solid-State Zn-Ion Batteries. *Adv. Mater.* **32**, 1908121 (2020).
7. Ma LT, Chen SM, Li XL, Chen AO, Dong BB, Zhi CY. Liquid-Free All-Solid-State Zinc Batteries and Encapsulation-Free Flexible Batteries Enabled by In Situ Constructed Polymer Electrolyte. *Angew Chem., Int. Ed.* **59**, 23836-23844 (2020).
8. Tang Y, *et al.* Ion-confinement effect enabled by gel electrolyte for highly reversible dendrite-free zinc metal anode. *Energy Storage Mater.* **27**, 109-116 (2020).
9. Cong JL, *et al.* Ultra-stable and highly reversible aqueous zinc metal anodes with high preferred orientation deposition achieved by a polyanionic hydrogel electrolyte. *Energy Storage Mater.* **35**, 586-594 (2021).
10. Zhang B, *et al.* Tuning Zn^{2+} coordination tunnel by hierarchical gel electrolyte for dendrite-free zinc anode. *Sci. Bull.* **67**, 955-962 (2022).
11. Liu Q, Wang Y, Hong X, Zhou R, Hou Z, Zhang B. Elastomer-Alginate Interface for High-Power and High-Energy Zn Metal Anodes. *Adv. Energy Mater.* **12**, 2200318 (2022).
12. Yang JL, Li J, Zhao JW, Liu K, Yang P, Fan HJ. Stable Zinc Anode Enabled by Zincophilic

Polyanionic Hydrogel Layer. *Adv. Mater.* **34**, 2202382 (2022).

13. Zhang W, *et al.* Kinetics-Boosted Effect Enabled by Zwitterionic Hydrogel Electrolyte for Highly Reversible Zinc Anode in Zinc-Ion Hybrid Micro-Supercapacitors. *Adv. Energy Mater.* **12**, 2202219 (2022).
14. Shao Y, *et al.* Regulating Interfacial Ion Migration via Wool Keratin Mediated Biogel Electrolyte toward Robust Flexible Zn-Ion Batteries. *Small* **18**, 2107163 (2022).
15. Zeng X, *et al.* Bio-inspired design of an in situ multifunctional polymeric solid-electrolyte interphase for Zn metal anode cycling at 30 mA cm^{-2} and 30 mA h cm^{-2}. *Energy Environ. Sci.* **14**, 5947-5957 (2021).
16. Wei T, Ren Y, Li Z, Zhang X, Ji D, Hu L. Bonding interaction regulation in hydrogel electrolyte enable dendrite-free aqueous zinc-ion batteries from −20 to 60 °C. *Chem. Eng. J.* **434**, 134646 (2022).
17. Hao Y, Feng D, Hou L, Li T, Jiao Y, Wu P. Gel Electrolyte Constructing Zn (002) Deposition Crystal Plane Toward Highly Stable Zn Anode. *Adv. Sci.* **9**, 2104832 (2022).
18. Zhou J, *et al.* Encapsulation of Metallic Zn in Hybrid MXene/Graphene Aerogel as Stable Zn Anode for Foldable Zn-ion Batteries. *Adv. Mater. Inter.* **34**, 2106897 (2021).
19. Chen M, Chen J, Zhou W, Han X, Yao Y, Wong CP. Realizing an All-Round Hydrogel Electrolyte toward Environmentally Adaptive Dendrite-Free Aqueous Zn-MnO_2 Batteries. *Adv. Mater.* **33**, 2007559 (2021).
20. Huang S, Hou L, Li T, Jiao Y, Wu P. Antifreezing Hydrogel Electrolyte with Ternary Hydrogen Bonding for High-Performance Zinc-Ion Batteries. *Adv. Mater.* **34**, 2110140 (2022).
21. Li M, *et al.* Comprehensive H_2O Molecules Regulation via Deep Eutectic Solvents for Ultra-Stable Zinc Metal Anode. *Angew Chem., Int. Ed.* **62**, 202215552 (2023).
22. Lin Y, Mai Z, Liang H, Li Y, Yang G, Wang C. Dendrite-free Zn anode enabled by anionic surfactant-induced horizontal growth for highly-stable aqueous Zn-ion pouch cells. *Energy Environ. Sci.* **16**, 687-697 (2023).
23. Zhang H, *et al.* Inducing the Preferential Growth of Zn (002) Plane for Long Cycle Aqueous Zn-Ion Batteries. *Adv. Energy Mater.* **13**, 2203254 (2022).

24. Yang X, Deng W, Chen M, Wang Y, Sun CF. Mass-Producible, Quasi-Zero-Strain, Lattice-Water-Rich Inorganic Open-Frameworks for Ultrafast-Charging and Long-Cycling Zinc-Ion Batteries. *Adv. Mater.* **32**, 2003592 (2020).

25. Chen Y, *et al.* Low Current‐Density Stable Zinc‐Metal Batteries Via Aqueous/Organic Hybrid Electrolyte. *Batteries Supercaps* **5**, 202200001 (2022).

26. Chen Z, *et al.* Polymeric Single Ion Conductors with Enhanced Side Chains Motion for High-performance Solid Zinc Ion Batteries. *Adv. Mater.* **34**, 2207682 (2022).

27. Ma G, *et al.* Reshaping the electrolyte structure and interface chemistry for stable aqueous zinc batteries. *Energy Storage Mater.* **47**, 203-210 (2022).
